# Differences in early risk factors for obesity between African American formula-fed infants and White breastfed controls

**DOI:** 10.1186/s40814-017-0198-8

**Published:** 2017-11-15

**Authors:** Paige K. Berger, Justin A. Lavner, Jessica J. Smith, Leann L. Birch

**Affiliations:** 1 Department of Preventive Medicine, University of Southern California, Los Angeles, CA USA; 2 Department of Psychology, University of Georgia, Athens, GA USA; 3 Department of Foods and Nutrition, University of Georgia, Athens, GA USA

**Keywords:** Obesity, Growth, Race/ethnicity, Prevention, Infancy

## Abstract

**Background:**

Previous RCTs to prevent early rapid weight gain were conducted in predominantly White, well-educated, middle-income mother-infants at low risk for obesity. To inform the design of an RCT in a higher-risk sample, we conducted a short-term, longitudinal study to compare maternal feeding beliefs and behaviors, infant sleep, intake, and growth of African American formula feeding (AAFF) dyads to a comparison sample of White breastfeeding (WBF) dyads. We also assessed the feasibility of recruiting and retaining AAFF participants.

**Methods:**

AAFF (*n* = 32) and WBF (*n* = 25) mother-infants were assessed at 2, 8, and 16 weeks postpartum. Data included demographics and maternal reports of feeding beliefs and behaviors, infant sleep, meal size, and feeding frequency, and measured infant length and weight.

**Results:**

AAFF and WBF mothers differed in demographics. AAFF mothers reported greater agreement with pressuring the infant to eat and feeding to soothe a fussy infant. Compared to WBF infants, AAFF infants slept fewer hours and consumed more grams/feeding from 2 to 16 weeks. There were no group differences in feeding frequency, which resulted in AAFF infants consuming more grams/day of milk than WBF infants. AAFF infants had lower gestational age, lower weight at 2 weeks, and had more rapid weight gain from 8 to 16 weeks.

**Conclusions:**

Findings point to potentially modifiable risk factors that may underlie disparities in early obesity among AAFF infants, including short sleep duration, feeding beliefs and behaviors, and rapid growth, but also confirm the challenges of recruiting and retaining AAFF participants, all of which inform the design and feasibility of an early preventive intervention.

**Trial registration:**

Retrospectively registered in clinicaltrials.gov on August 23, 2016 (2013102510).

## Background

Infancy is a critical period of developmental plasticity, and rapid weight gain in infancy is an early risk factor for obesity later in life [1]. Previous randomized controlled trials (RCT) have reduced rapid weight gain by providing mothers with responsive parenting (RP) guidance [2, 3]. Mothers were taught to minimize the use of feeding for non-hunger-related fussiness, recognize and respond to infant hunger and fullness cues to allow the infant to determine how much is consumed, and to foster infant self-soothing to sleep, rather than feeding to sleep [4]. Compared to a safety control, RP infants grew more slowly and had lower weight status at 1 year [3, 5], and had longer night sleep durations [4]. However, these trials were conducted in relatively low-risk samples. The first RCT included only breastfeeding mothers [3], while the second included breastfeeding and formula feeding mothers [2, 5]; in both, participants were predominantly from non-Hispanic White, well-educated, middle-income families.

To inform the development of a similarly efficacious intervention for a higher risk sample, we recruited a sample of African American mother-infant dyads. African American women are more likely to formula-feed than breastfeed [6, 7]. Formula feeding can be a risk factor for obesity; it is associated with more rapid weight gain from 2 to 12 months postpartum [8]. Differences in patterns of growth may also be due to differences in sleep, meal size, or feeding frequency; breastfeeding infants tend to be fed more frequently, while formula feeding infants tend to consume larger volumes per feeding [9]. Given the ethnographic evidence that African American women engage in infant feeding practices that encourage excessive intake [10, 11], providing early guidance on RP could reduce obesity risk by supporting more normative infant growth [6–8, 10–12].

The primary aim of this short-term, longitudinal study was to inform the design of an RCT for early obesity prevention among a high-risk sample by obtaining information on several potentially modifiable risk factors for rapid growth among low-income African American formula feeding (AAFF) dyads. This higher risk group was compared to a sample of White breastfeeding (WBF) dyads, similar to those participating in our previous RCTs [2, 3] and to the WHO sample of exclusively breastfeeding infants who were the growth “standard” for the revised CDC growth charts [13]. We examined differences in maternal feeding beliefs and behaviors, infant total and nighttime sleep, feeding size, and feeding frequency. To assess differences in rapid weight gain, we measured infant growth parameters at 2, 8, and 16 weeks postpartum, when differences in growth rates begin to emerge [8, 14]. We also assessed the feasibility of recruiting and retaining low-income AAFF participants.

## Methods

### Subjects

Participants were recruited beginning in July of 2014 using targeted direct mail and email campaigns, advertisements on local mass transit, along with brochure placement in obstetric practices, in facilities offering low-cost prenatal care, and in the area’s Special Supplemental Nutrition Program for Women, Infants, and Children (WIC) clinic. Trained study personnel administered telephone screens and explained that the purpose of the study was “to assess how type and amount of infant feeding affect the infant’s growth.” Mothers were included in the study if they were 18 to 45 years old and were currently ≥ 12 weeks pregnant or had a newborn (≤ 28 days, ≥ 37 weeks gestation, birth weight ≥ 2.5 kg). Exclusion criteria included: a diagnosis of gestational diabetes, hypertension, or other existing medical condition that may affect healthy delivery and infant weight, as well as any prescription medications known to influence weight. Data collection was completed in July of 2016.

Feeding mode was determined by self-report at 2 weeks postpartum and monitored by questionnaire at 8 weeks postpartum and confirmed by feeding logs. To categorize dyads by feeding mode, we used the criteria developed for the CDC’s Infant Feeding Practices II Survey [15]; infants were considered predominantly breastfed when ≥ 80% of feedings were breast milk and predominantly formula-fed when ≥ 80% of feedings were formula. To minimize the heterogeneity within groups and because our aim was to compare modifiable risk factors for obesity in AAFF dyads to WBF dyads, those not meeting the criteria for either formula feeding or breastfeeding were excluded from the study analyses.

### Design and procedures

Data were collected at 2, 8, and 16 weeks postpartum. Mothers completed questionnaires, and maternal and infant anthropometric data were obtained during scheduled visits to the Child Nutrition Laboratory at The University of Georgia.

### Measures

#### Infant feeding style

Infant feeding beliefs were assessed at the 8-week visit using the Infant Feeding Style Questionnaire (IFSQ) maternal feeding beliefs questions [16]. Given the focus of our prior RCTs, which promoted the use of responsive feeding practices (e.g., the use of hunger and fullness cues to determine how much to feed and minimizing the use of feeding to soothe for non-hunger-related fussiness), we included measures of maternal beliefs regarding pressure to eat and feeding to soothe a fussy infant. The pressure subscale consisted of five items, including “it’s important for an infant to finish all of the milk in his/her bottle.” The feeding to soothe subscale consisted of three items, including “when an infant cries it usually means he/she needs to be fed.” Responses were measured on a 5-point Likert-type scale, ranging from disagree (1) to agree (5). Mothers also answered questions about whether they had offered their infant other beverages or solid foods and about adding cereal to the bottle [2].

#### Infant sleep

Infant sleep was assessed at the 16-week visit using questions from the Brief Infant Sleep Questionnaire (BISQ) [17]. Mothers reported the number of total sleep hours, nighttime sleep hours, and nighttime sleep waking on a typical day in the past week. Mothers reported how often infants had difficulty self-soothing to return to sleep, and how often feeding was used to return the infant to sleep.

#### Feeding logs

Mothers completed six 24-h infant feeding logs. Trained study personnel provided instructions on how to complete the logs. Mothers were asked to record the time of each feeding episode for one full 24-h period at 2, 4, 6, 8, 10, and 12 weeks postpartum. Formula feeding mothers recorded the amount of formula in the bottle (in ounces) before and after each feeding. Breastfeeding mothers used an electronic infant scale (Medela BabyWeigh II; Medela Inc., McHenry, IL, USA) and were instructed on how to weigh the infant and to record the infant’s weights (in grams) before and after each feeding. Mean grams per feeding and mean feedings per day were calculated for two separate periods: between 2- and 8-week visits, using 2-, 4-, and 6-week logs, and between 8- and 16-week visits, using 8-, 10-, and 12-week logs. Dyads with at least one completed feeding log during the two periods were included in the analysis.

#### Anthropometrics

Infant recumbent length was measured in duplicate using an infantometer to the nearest 0.1 cm (Seca 416; Seca Corp, Birmingham, United Kingdom). Infant weight was measured with no clothing using an electronic scale to the nearest 0.1 kg (PEA POD; Life Measurement Inc., Concord, CA, USA). CDC does not recommend the use of BMI for children under 24 months. Weight-for-age *z*-scores were chosen because they adjust for differences in infant age at time of visit and were calculated using WHO growth charts [18].

### Statistical analyses

#### Power calculation

A post hoc reverse power calculation was completed using G Power 3.1.9.2. A total sample size of 32 formula feeding infants and 25 breastfeeding infants with an alpha level of 0.05 (two-tailed) had adequate power to detect a large effect size (*d* = 0.80) between groups using *t* tests (power = 0.84). Achieved power for a medium effect (*d* = 0.50) was 0.45.

#### Analyses

Data were analyzed using SPSS software (version 22.0, 2013; IBM SPSS Statistics, Armonk, NY). Each outcome variable was assessed for normality. Group differences in participant characteristics, maternal reports of pressure and feeding to soothe beliefs on the IFSQ, maternal reports of sleep hours, night waking, self-soothing, and feeding to return to sleep on the BISQ, and maternal reports of meal size and feeding frequency were examined using *t* tests and ANOVA and chi-square tests.

We analyzed infant weight-for-age at each time point and growth over time using weight-for-age *z*-scores; however, for ease of interpretation, we present corresponding weight-for-age percentiles. We tested whether there were differences between the AAFF sample and WBF sample in change in weight-for-age *z*-scores from (1) 2 to 8 weeks and (2) 8 to 16 weeks using linear regression analysis; weight-for-age *z* at the follow-up assessment was regressed on race/feeding mode, weight-for-age *z* at the previous assessment, and adjusting for time between assessments. *P* values < 0.05 were considered significant.

## Results

**Fig. 1:**
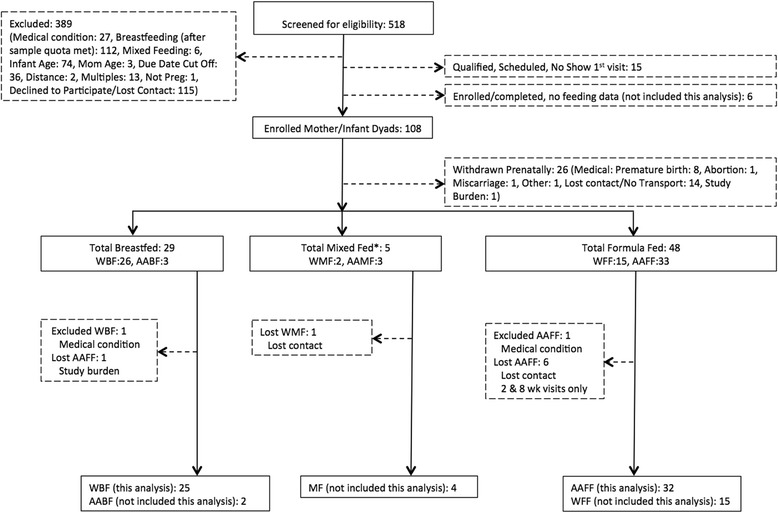
CONSORT flow diagram of study participants

As shown in Fig. 1, of the 518 women screened for eligibility, 114 qualified and were enrolled in the study. Of those, 108 mother-infant dyads are reported upon here. Twenty-six women (~ 23%) were withdrawn prenatally, most commonly due to premature birth (8) or to loss of contact/transportation issues (14). The majority of women who withdrew prenatally (81%) were African American, with abortion, late-term miscarriage, and study burden cited as reasons for discontinuation. Excluded from this analysis were five subjects who did not meet the criteria for predominantly breast or formula feeding. Among the 59 remaining AAFF and WBF participants, two were excluded from this analysis due to an infant medical condition. Of the 57 included in this analysis, 51 completed the study, representing a retention rate of 89%. Attrition was mainly due to loss of contact prior to 16 weeks with six AAFF mothers.

Participant characteristics presented in Table 1 indicate that compared to WBF mothers, AAFF mothers had higher BMIs, were significantly younger, had lower levels of education and income, and a higher proportion of WIC participation. AAFF infants had significantly lower gestational age at birth, weight at 2 weeks, and length at 2 weeks than WBF infants.

**Table 1:** Participant characteristics for African American formula feeding (AAFF) and White breastfeeding (WBF) mother-infants^a^

	AAFF (*n* = 32)	WBF (*n* = 25)
Mothers		
Age, years^**^	24.6 (0.7)	32.7 (0.7)
Weight, kg^**^	85.8 (2.9)	71.6 (3.1)
BMI, kg/m2^**^	32.6 (1.1)	26.3 (1.2)
Parity	2.31 (0.2)	1.72 (0.2)
Education, %^b,**^		
High school graduate or less	90.3	14.3
Some college/technical school	3.20	33.3
College graduate or more	3.20	52.4
Income, %^b,**^		
< $25,000	45.2	9.52
$25,000–49,999	6.45	23.8
$50,000–74,999	9.68	23.8
≥ $75,000	3.23	42.9
WIC participation, %^b,**^	86.2	23.8
Infants		
Gestational age, weeks^**^	39.1 (0.2)	40.1 (0.2)
Age, weeks^*^	2.45 (0.1)	2.01 (0.1)
Male/female, %^b^	50/50	36/64
Weight, kg^*^	3.46 (0.1)	3.72 (0.1)
Length, cm^**^	49.5 (0.4)	51.5 (0.4)

Values are mean (SE) or %

*P < 0.05; **P < 0.01

^a^Tests of significance between groups were based on ANOVA unless otherwise indicated

^b^Tests of significance between groups were based on the chi-square test of goodness of fit

There were consistent significant group differences in maternal feeding beliefs and behaviors. On the IFSQ, AAFF mothers reported greater agreement with the use of pressuring the infant to eat [2.91 (SE = 0.17) vs. 1.55 (SE = 0.19); *P* = 0.000] and using feeding to soothe a fussy infant [3.32 (SE = 0.19) vs. 2.45 (SE = 0.20); *P* = 0.003] than WBF mothers. By 8 weeks, more than a quarter of AAFF mothers reported putting cereal in the bottle [(28 vs. 0%, *χ* ^2^ (1, *N* = 47) = 5.94, *P* = 0.01)].

**Fig. 2:**
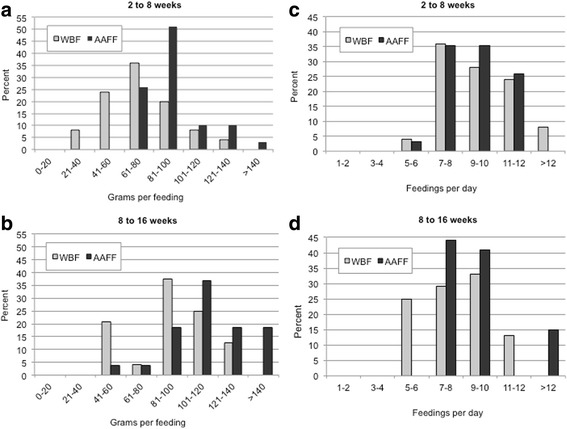
Distribution of mean grams/feeding, mean feedings/day: African American formula feeding (AAFF) and White breastfeeding (WBF) infants. **a** Grams/feeding, 2 to 8 weeks. **b** Grams/feeding, 8 to 16 weeks. **c** Feedings/day, 2 to 8 weeks. **d** Feedings/day, 8 to 16 weeks. Tests of significant differences between groups were based on ANOVA (*P* < 0.05). AAFF infants consumed more grams per feeding from 2 to 8 weeks [92.32 (SE = 3.35) vs. 71.44 (SE = 4.99); *P* = 0.001)] and from 8 to 16 weeks [115.15 (SE = 5.08) vs. 91.50 (SE = 4.87); *P* = 0.002] than WBF infants

As shown in Fig. 2, AAFF infants consumed significantly more grams per feeding than WBF infants from 2 to 8 weeks (Fig. 2a), and from 8 to 16 weeks (Fig. 2b). There were no significant differences between AAFF and WBF infants in the number of feedings per day from 2 to 8 weeks [9.08 (SE = 0.38) vs. 9.54 (SE = 0.39); *P* = 0.399] (Fig. 2c) or from 8 to 16 weeks [9.04 (SE = 0.33) vs. 8.33 (SE = 0.34); *P* = 0.166] (Fig. 2d). AAFF infants also slept significantly fewer total hours [11.43 (SE = 0.53) vs. 12.95 (SE = 0.51); *P* = 0.047] and nighttime hours [7.33 (SE = 0.39) vs. 9.50 (SE = 0.38); *P* = 0.000] than WBF infants. There were no significant differences between AAFF and WBF infants in nighttime sleep-waking, self-soothing to return to sleep, or feeding to return to sleep.

**Fig. 3:**
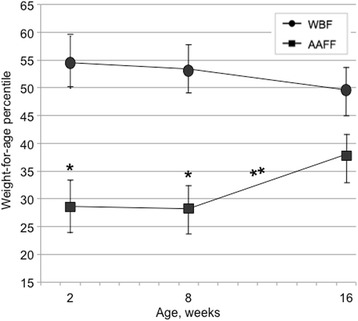
Weight-for-age percentiles over time: African American formula feeding (AAFF) and White breastfeeding (WBF) infants. Tests of significance between groups were based on linear regression (*P* < 0.05). *AAFF infants had lower weight-for-age *z*-scores relative to WBF infants at 2 weeks [−0.68 (SE = 0.14) vs. 0.14 (SE = 0.15); *P* = 0.000] and 8 weeks [−0.72 (SE = 0.16) vs. 0.09 (SE = 0.13); *P* = 0.000]. **AAFF infants had significantly greater increases in weight-for-age *z* from 8 to 16 weeks than WBF infants (Beta = 0.17, *t* = 2.13, *P* = 0.04)

AAFF infants had significantly lower weight-for-age *z*-scores relative to WBF infants at 2 and 8 weeks, but not at 16 weeks. This was due to AAFF infants having significantly greater increases in weight-for-age *z* from 8 to 16 weeks than WBF infants. Figure 3 shows these different patterns of change from 8 to 16 weeks, as mean weight-for-age percentile for AAFF infants increased during this period from the 28th percentile to the 37th percentile, while mean weight-for-age percentile for WBF infants continued to track near the 50th percentile.

## Discussion

This pilot study describes differences in maternal feeding attitudes and practices that may underlie disparities in risk for obesity in low-income AAFF infants that may be used as specific targets for intervention. Compared to WBF mothers, AAFF mothers were much more likely to subscribe to feeding beliefs and behaviors that promote excessive intake; they reported greater agreement with pressuring the infant to eat and using feeding to soothe. AAFF mothers also reported shorter total and nighttime sleep durations among their infants. AAFF infants consumed significantly more milk per feeding, but surprisingly did not differ from WBF infants in the number of feedings per day. AAFF infants also showed evidence of “catch up” growth, having more rapid weight gain than WBF infants between 8 and 16 weeks postpartum. Because results of our previous RCTs indicate that soothing, sleeping, and feeding practices are modifiable and underlie early risk for obesity, these patterns suggest that AAFF dyads may benefit from an RP intervention to promote healthy growth [3–5]. Recruitment and retention of AAFF dyads proved to be more challenging than recruitment and retention of WBF dyads, which is valuable information to be incorporated in the design of a subsequent trial.

In the USA, the prevalence of formula feeding is substantially higher among African Americans than among other racial/ethnic groups [6, 7]. Formula feeding can be a risk factor for childhood obesity, particularly when it occurs in conjunction with other feeding beliefs, such as pressuring the infant to “finish the bottle” [15, 19] and that feeding is the best way to soothe a fussy infant, or behaviors, such as adding cereal to the bottle and the early introduction of solids [6]. Our current findings show a similar constellation of infant feeding beliefs and behaviors associated with formula feeding among our AAFF sample, although few mothers in either group reported introducing solid foods, which was asked at 8 weeks but not at 16 weeks.

Although these feeding practices tend to be used in the belief that they promote infant sleep (as indicated in items on the IFSQ), we found that AAFF infants slept fewer total hours and nighttime hours than WBF infants at 16 weeks postpartum. According to the National Sleep Foundation, it is recommended that infants at this age sleep 12 to 15 h per day [20]. On average, AAFF infants were not meeting the minimum recommendation; AAFF infants slept ~ 11.4 h per day, while in contrast, WBF infants were within the suggested range, sleeping an average of 13.0 h per day. These results are consistent with shorter sleep durations reported among African American children [21]. Shorter sleep duration is a risk factor for obesity throughout the lifespan, including infancy and early childhood [21, 22]. Infants often wake due to hunger or other distress. This is responded to with more frequent feedings, resulting in shorter sleep bouts [23] and less time spent sleeping, which can increase infant fussiness and prompt additional feedings [24].

Consistent with this, AAFF infants had greater total daily milk intake than WBF infants. AAFF infants had larger feedings than WBF infants, similar to previous reports of greater grams per feeding for formula-fed than breastfed infants [9]. In our study, there were no differences in feeding frequency by feeding mode. However, between 8 and 16 weeks, the majority of WBF infants were fed 7 to 12 times per day, consistent with guidance from the American Academy of Pediatrics (AAP) (i.e., 8 to 12 times per day). AAFF infants were also reportedly fed 7 to 12 times per day, much more frequently than is recommended by the AAP for formula-fed infants (i.e., 5 to 6 times per day) [9]. These patterns suggest that AAFF dyads could benefit from RP guidance to reduce overfeeding, which could include discriminating infant hunger from other sources of distress and recognizing infant fullness cues [3–5].

We observed differences in growth patterns for AAFF and WBF infants. AAFF infants had a lower gestational age at birth and at 2 weeks postpartum, and had a mean weight-for-age at the 30th percentile, compared to WBF infants who tracked just above the 50th percentile. Between 8 and 16 weeks, AAFF infants gained weight more rapidly than WBF infants. This is generally consistent with both the “catch-up” growth seen among infants with lower birth weights and evidence that by 3 to 4 months, formula-fed infants are growing more rapidly than breastfed infants [8]. A recent study revealed that among African American children, those born at a low weight percentile and who had rapid growth in the first 6 months postpartum also had the highest prevalence of obesity at age 5 years [25]. Our AAFF infants showed this pattern of vulnerability, underscoring their higher risk for obesity, and that low-income AAFF dyads, in particular, may benefit from an intervention affecting soothing, sleeping, and feeding to alter this growth trajectory.

This study is not without limitations. We relied on maternal reports, which are subject to error and reporting bias, and used different methods for collecting feeding data for breastfeeding and formula-feeding infants; this could have contributed to the differences between groups, particularly in amounts consumed. In this small sample, multiple comparisons increase the risk for type 1 error and power is limited to detect effects. However, despite limited power, these findings show a coherent pattern of significant differences between AAFF and WBF dyads in maternal feeding attitudes, practices, and infant sleeping, feeding, and growth that may increase disparities in early risk for obesity. A larger RCT might also measure the extent that mothers enforced feeding beliefs and behaviors (e.g., bottle emptying).

Finally, findings indicate that issues of recruitment, retention, participant burden, and culturally specific factors will need to be addressed in tailoring an intervention for AAFF dyads. Both recruitment and retention of AAFF mothers were more difficult than that of WBF mothers (e.g., more time and funding spent on recruitment efforts, more women excluded during screening, and more frequent premature birth or loss of contact before delivery due to medical complications). Mothers were compensated for participation via institutional check request delivered by mail after each visit. They received $60 at the 2- and 8-week visits and $80 at the 16-week visit. Feeding logs were compensated for a total of up to $150 (six logs, $25 each). Although the compensation schedule was rated overall to be satisfactory to participants, processing of checks was time intensive. This could be addressed by offering compensation at the end of each appointment to help increase attendance and retention. Frequent changes in living arrangements and cell phone numbers as well as a lack of access to transportation for appointments were obstacles to scheduling and retaining AAFF dyads. Although we could not provide transportation due to time and staffing constraints, doing so might attenuate subject loss in future studies. We could also use home visits, as we have done in our previous research [2]. These have been shown to have lasting favorable effects on long-term social and health outcomes, and may be an option to reduce study attrition [26].

Overall, our results suggest that an RP intervention designed for AAFF dyads to reduce rapid growth and early obesity could be efficacious. However, the findings also indicate that maternal beliefs about infant feeding that are used to inform and motivate their parenting practices may be a barrier to change. This will also need to be considered in the development of future studies for this higher risk sample.

## Conclusions

Findings from this small pilot study revealed that AAFF and WBF dyads differed in many ways, including early growth rate. In addition, groups differed in demographics; gestational age, weight, and length; maternal feeding beliefs and behaviors; and infant sleep, meal size, and feeding frequency. The patterns observed among AAFF dyads, who reported greater agreement with pressure and feeding to soothe practices, fewer infant sleep hours, and greater total daily milk intakes, have been previously shown to increase early risk for obesity. Our data provide some evidence that an RP intervention, which has affected change in parenting practices among White middle-income samples, could be efficacious for this higher risk AAFF sample [3, 5].

## Abbreviations

AAFF: African American formula feeding
BISQ: Brief Infant Sleep Questionnaire
IFSQ: Infant Feeding Style Questionnaire
RCT: Randomized controlled trial
RP: Responsive parenting
WBF: White breastfeeding
WIC: Special Supplemental Nutrition Program for Women, Infants, and Children
